# Removal Mechanism Investigation of Ultraviolet Induced Nanoparticle Colloid Jet Machining

**DOI:** 10.3390/molecules26010068

**Published:** 2020-12-25

**Authors:** Xiaozong Song, Gui Gao

**Affiliations:** 1 School of Mechanical and Electronical Engineering, Lanzhou University of Technology, Lanzhou 730050, China; 2 State Key Laboratory of Solid Lubrication, Lanzhou Institute of Chemical Physics, Chinese Academy of Sciences, Lanzhou 730000, China; gaogui@licp.cas.cn

**Keywords:** first-principles simulation, nanoparticles, adsorption, ultra-smooth surface

## Abstract

Ultraviolet induced nanoparticle colloid jet machining is a new ultra-precision machining technology utilizing the reaction between nanoparticles and the surface of the workpiece to achieve sub-nanometer ultra-smooth surface manufacturing without damage. First-principles calculations based on the density functional theory (DFT) were carried out to study the atomic material removal mechanism of nanoparticle colloid jet machining and a series of impacting and polishing experiments were conducted to verify the mechanism. New chemical bonds of Ti-O-Si were generated through the chemical adsorption between the surface adsorbed hydroxyl groups of the TiO_2_ cluster and the Si surface with the adsorption energy of at least −4.360 eV. The two Si-Si back bonds were broken preferentially and the Si atom was removed in the separation process of TiO_2_ cluster from the Si surface realizing the atomic material removal. A layer of adsorbed TiO_2_ nanoparticles was detected on the Si surface after 3 min of fixed-point injection of an ultraviolet induced nanoparticle colloid jet. X-ray photoelectron spectroscopy results indicated that Ti-O-Si bonds were formed between TiO_2_ nanoparticles and Si surface corresponding to the calculation result. An ultra-smooth Si workpiece with a roughness of Rq 0.791 nm was obtained by ultraviolet induced nanoparticle colloid jet machining.

## 1. Introduction

**Figure 1:**
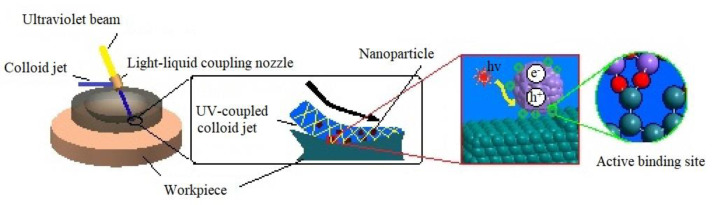
Schematic diagram of ultraviolet induced nanoparticle colloid jet machining.

At present, ultra-smooth surfaces with high precision surface figure accuracy and extremely low surface roughness have been widely used in optics, electronic science and other fields, and have urgent needs in various fields [1,2,3]. In order to manufacture the ultra-smooth surface without damage, a variety of surface processing technologies and methods have been developed [4,5,6]. Chemical mechanical polishing is a kind of machining method to remove workpiece surface material by chemical mechanical interaction. However, it is mainly used for planarization of flat workpieces [7,8,9,10]. High energy beam processing technology uses high density energy beams (ion beam, electron beam, laser beam, etc.) to remove the surface material of a workpiece with a relatively high processing cost [11]. Magnetorheological polishing technology can achieve high-precision controllable polishing [12,13], the surface roughness after processing is about Ra 1nm. Fluid jet polishing utilizes the erosion effect of abrasive particles in a high-speed jet to remove the workpiece surface material, which can greatly improve the surface roughness and surface morphology [14], but defects such as tiny cracks may be introduced onto the workpiece surface. Laser assisted water jet micromachining uses the photo thermal effect of a high-energy laser beam to heat and soften the surface of hard and brittle materials, and then an abrasive water jet beam is used to slightly remove the softened area [15,16]. In the elastic emission machining, micro and nano abrasives in polishing fluid react with the workpiece surface under the action of a hydrodynamic pressure field in the micron scale gap between the polishing tool and the workpiece surface to remove the nano scale materials on the workpiece surface and can obtain super smooth surface. Elastic emission machining technology has high requirements for processing environment and machine tool stability [17]. Ultraviolet induced nanoparticle colloid jet machining is a new polishing technology which utilizes the interaction between nanoparticles in colloid and workpiece surfaces under the irradiation of catalytic light to remove the workpiece surface material at the atomic level and obtain an ultra-smooth surface, as is shown in Figure 1. The mechanism of surface interaction between nanoparticles and the workpiece surface in ultraviolet induced nanoparticle colloid jet machining has not been fully presented so far. How the hydroxyl groups in colloid act as active binding sites and form the connection between nanoparticles and the workpiece surface needs to be further studied. The quantification, investigation and knowledge of the behavior of nanoparticles in the colloid under ultraviolet radiation can help to properly understand the material removal mechanism in ultraviolet induced nanoparticle colloid jet machining.

## 2. Calculation Method and Model

All density functional theory (DFT) calculations in this study were performed by using the CASTEP (Cambridge Sequential Total Energy Package) program [18,19] with Vanderbilt-type ultrasoft pseudo potentials [20] and a plane–wave expansion of the wave functions. The Perdew–Burke–Ernzerhof (PBE) exchange correlation functional under generalized gradient approximation (GGA) was used as the exchange-associative interaction, and the ultrasoft pseudopotential (USPP) [21] was used to describe the interaction between electrons and ions. The cut-off energy was set as 500 eV for the TiO_2_ cluster and 300 eV for the Si surface system, respectively. The sample of the Brillouin zone K point was 3 × 3 × 1. The geometries of the system were relaxed until the residual forces were less than 0.05 eV/Å, the stress was less than 0.1 GPa, the total energy was less than 2.0 × 10^−5^ eV/atom and the change in displacement was less than 0.002 Å. Spin polarization was not considered in the calculation.

**Figure 2:**
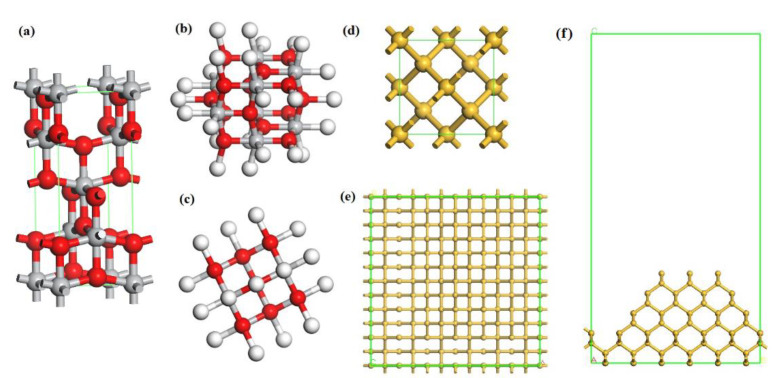
Calculated structures of anatase TiO_2_ cluster model and monocrystalline silicon surface. (**a**) Unit cell of TiO_2_, (**b**) Top view, and (**c**) side view of TiO_2_ cluster where white, red and grey are hydrogen, oxygen and titanium atoms, respectively; (**d**) Unit cell of Si; (**e**) Top view, and (**f**) side view of Si (100) surface.

In the process of ultraviolet induced nanoparticle colloid jet machining, anatase titanium dioxide colloid was compounded as the polishing agent. A TiO_2_ cluster model (Ti_8_O_14_H_26_) and silicon supercell were set up to analyze the adsorption mechanism of TiO_2_ nanoparticles on the silicon workpiece surface in ultraviolet induced nanoparticle colloid jet machining, as is shown in Figure 2.

The calculated band gap of the TiO_2_ unit cell was 2.282 eV, which was smaller than the experimental value of 3.2 eV. However, since the band gap calculated by the GGA method is always less than the actual value, it is feasible to use the calculated data for comparison. The lattice constants of the anatase titanium dioxide model were a = b = 3.776 Å, c = 9.486 Å. The structure of anatase titanium dioxide was optimized, and the optimized Ti-O bond lengths were 1.930 Å and 1.973 Å, which were very similar to the experimental data 1.934 Å and 1.980 Å. The dangling bonds of the TiO_2_ cluster were saturated with H. The calculated band gap of the Si unit cell was 1.099 eV, which was smaller than the experimental data (about 1.2 eV). Considering the actual profile of the workpiece surface, an irregular convex Si (100) surface with periodic boundary conditions was set up as shown in Figure 2e,f. The Si surface was cut out from a Si 4 × 4 × 3 supercell. The lattice parameters of the silicon supercell were as follows: a = b = 23.04 Å, c = 44.93 Å, and the vacuum layer thickness was 30.0 Å. All the above models were optimized before calculation.

## 3. Calculation Results and Discussion

### 3.1. Adsorption of Hydroxyl Group on the Surface of Titanium Dioxide Cluster and Monocrystalline Silicon

**Figure 3:**
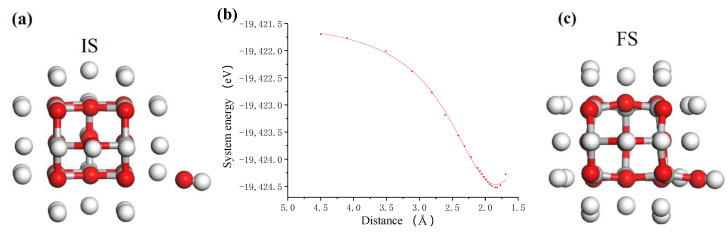
The process of OH group adsorbing on TiO_2_ cluster. (**a**) Initial state (IS); (**b**) system energy; (**c**) final state (FS) where white, red and grey balls are hydrogen, oxygen and titanium atoms, respectively.

A large number of hydroxyl (OH) groups can be produced in the TiO_2_ colloid under the action of photocatalysis of ultraviolet light [22]. These OH groups can interact with the unsaturated atoms on the surface of the nanoparticles and the workpiece to achieve surface adsorption and generate surface hydroxyl groups. Figure 3 shows the adsorption process of OH group on a suspended unsaturated Ti atom of TiO_2_ cluster. Figure 3b shows the relationship between the total system energy and the distance in the adsorption process. According to the intensity of the curve variation, it is considered that the interaction between OH group and TiO_2_ cluster changes from a weak interaction to a strong interaction when the distance is about 3.0 Å, and the interaction becomes repulsive when the distance is about 1.8 Å. Therefore, it can be considered that the OH is adsorbed onto the TiO_2_ cluster and bonded at this point. In the colloid, the process of OH group adsorbing on the TiO_2_ nanoparticle is an exothermic process with the adsorption energy of about 1.87 eV. After bonding, the structure of the system was optimized, and the final structure of the adsorption system was obtained, as shown in Figure 3c. The OH group in the colloid finally adsorbs on the target Ti atom with O atom end inward, and becomes the surface adsorbed hydroxyl group with Ti-O bond 1.794 Å.

**Figure 4:**
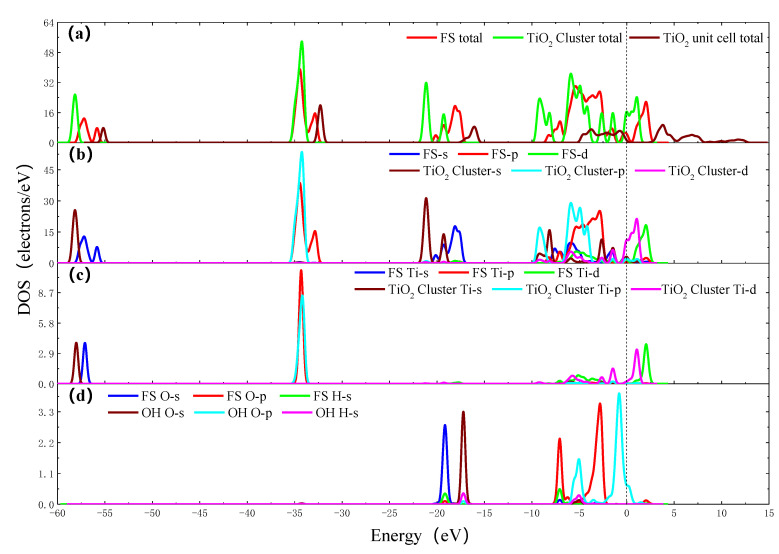
The density of states (DOS) of OH group adsorbing on the surface of TiO_2_ cluster. (**a**) Total DOS of FS, TiO_2_ cluster and TiO_2_ unit cell; (**b**) Partial density of states (PDOS) of FS and TiO_2_ cluster; (**c**) PDOS of adsorption site Ti atoms of FS and TiO_2_ cluster; (**d**) PDOS of O atom and H atom of FS and OH group.

The density of states (DOS) is important in further understanding the reaction process. Figure 4a shows the total DOS of TiO_2_ unit cell, TiO_2_ cluster (Ti_8_O_14_H_25_) and final state (FS) (Ti_8_O_15_H_26_). It can be seen from the figure that, compared with the total DOS of the TiO_2_ unit cell, the DOS of the FS (Ti_8_O_15_H_26_) moves downward, that is, the conduction band position is more negative, while the conduction band and valence band position of TiO_2_ cluster (Ti_8_O_14_H_25_) move more and more significantly. It is considered that the TiO_2_ cluster (Ti_8_O_14_H_25_) contains two oxygen vacancies, and the FS (Ti_8_O_15_H_26_) contains one oxygen vacancy. The oxygen vacancy impurity anatase is equivalent to the doped semiconductor. The higher the concentration of oxygen vacancies, the more electrons enter the conduction band, and the more negative shifts of conduction and valence band positions are [23]. It can be seen from Figure 4, that the Ti 4s orbit shifts to the right after the OH group adsorbing on the surface of TiO_2_ cluster, the energy level increases and the electronic activity increases. The electron strength of the Ti 3p orbit decreases. The Ti 3d orbit shifts to the right and the electron strength decreases. The O 2s, O 2p and H 1s orbits shift to the left, and the energy level decrease. In the −7~0 eV section, hybridization occurs between the O 2p and the Ti 3d orbits. In the above process, the electrons are redistributed, the O 2p and Ti 3d orbits obtain electrons, and the Ti 4S and the Ti 3p orbits lose electrons, and a chemical bond is formed between Ti and O atoms.

**Figure 5:**
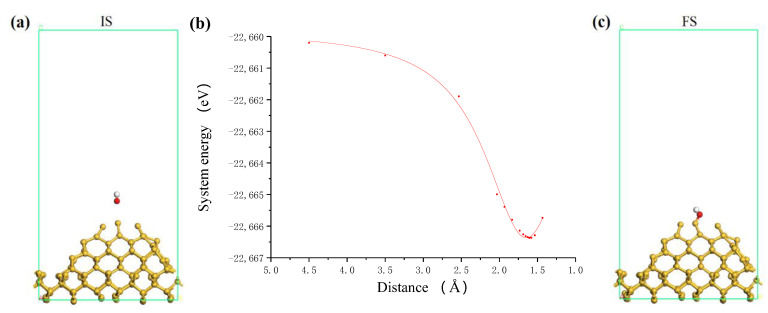
The process of OH group adsorbing on Si surface. (**a**) Initial state; (**b**) system energy; (**c**) final state where white and balls are hydrogen and oxygen atoms, respectively.

According to the calculation model, many unsaturated dangling bonds exist on the Si surface, so there are many possible bonding sites for the OH group. In the process of ultraviolet induced nanoparticle colloid jet machining, the location of impacting nanoparticles on the Si surface is random in principle. However, considering the influence of the actual workpiece surface morphology, the impacting nanoparticles will interact with the atoms at the highest position of Si surface firstly. Therefore, in the calculation of this paper, we chose a Si atom with the highest position on the Si surface as the target Si atom to study. Similar to the above process, the adsorption process of the OH group on the Si surface is shown in Figure 5. In this process, the O atom of the OH group is always close to the fixed direction of the target Si atom. According to the relationship curve of Figure 5b, the interaction between the OH group and the Si surface changes from a weak interaction to a strong interaction when the distance is about 2.6 Å, and the interaction becomes repulsive when the distance is about 1.65 Å. Again, it is considered that the OH is adsorbed on the Si surface and bonded with the target Si atom at this point. Because there is a large number of dangling bonds without passivation on the Si surface, it is easy enough for the OH group to adsorb on the Si surface and the adsorption energy is about 4.47 eV. Figure 5c shows the optimized final structure of the system after bonding. The OH group becomes a surface adsorbed hydroxyl group with Si-O bond 1.656 Å.

**Figure 6:**
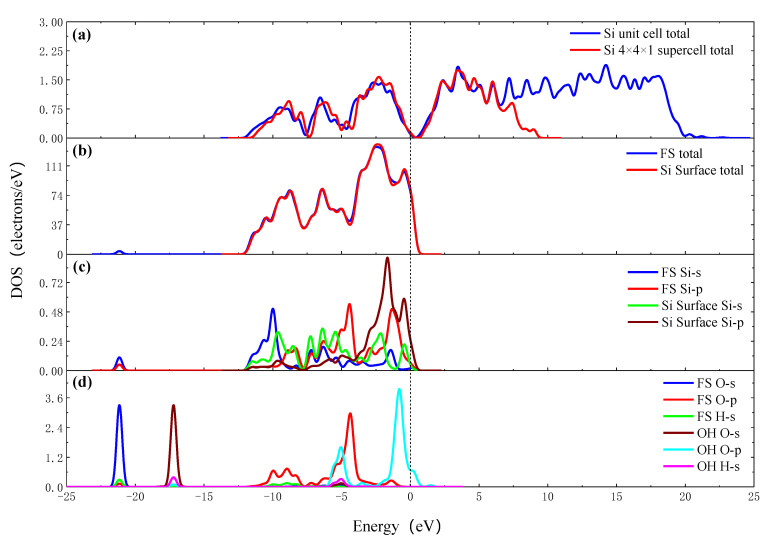
The DOS of OH group adsorbing on the Si surface. (**a**) Total DOS of Si unit cell and 4 × 4 × 1 supercell; (**b**) total DOS of FS and Si surface; (**c**) PDOS of adsorption site Si atoms of FS and Si surface; (**d**) PDOS of O atom and H atom of FS and OH group.

Figure 6 shows the density of states of the OH group adsorbing on the Si surface. Figure 6a,b shows the total DOS of the Si unit cell, Si 4 × 4 × 1 supercell, the Si surface total and the FS total. The valence band of the total DOS of the Si 4 × 4 × 1 supercell is similar to that of the Si unit cell, but the conduction band is greatly reduced. The Si surface, which is cut out from a Si 4 × 4 × 3 supercell, has a similar valence band peak with Si 4 × 4 × 1 supercell, while the conduction band is further reduced compared with Si 4 × 4 × 1 supercell. The valence band and conduction band of the Si surface move down simultaneously, and the Fermi surface of the Si surface enter the conduction band. It is considered that the Si surface has a large surface area and a large number of dangling bonds without passivation, there are more electrons in the higher energy quantum states, the surface states of the dangling bonds seriously affect the distribution of the DOS of the Si surface. The results of the PDOS show that the Si 3s orbit shifts to the left and the electron strength decreases. The Si 3p orbit shifts to the left and the electron strength increases. The O 2s, H 1s, orbits shift to the left from −17.25 eV and hybridizes with Si 3s and Si 3p orbits at −21.17 eV. In the −10~0 eV section, hybridization occurs between the O 2p, Si 3p and Si 3s orbits. The electrons are redistributed after OH group adsorbing on the Si surface. The O 2p, O 2s and Si 3p orbits get electrons, the Si 3s orbit lose electrons, and a chemical bond is formed between Si and O atoms.

### 3.2. Adsorption of Titanium Dioxide Cluster on the Silicon Surface

**Figure 7:**
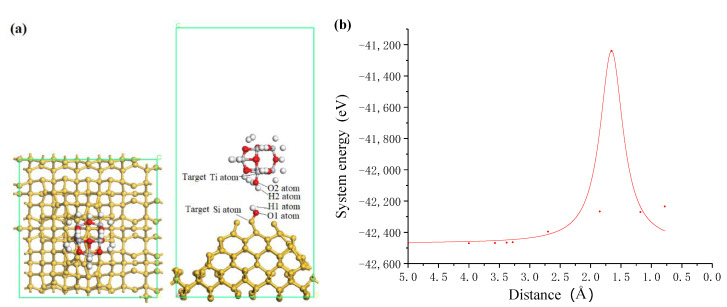
The incident impacting TiO_2_ cluster approaching to silicon surface. (**a**) Schematic diagram of incident state where white, red, grey and yellow balls are hydrogen, oxygen, titanium and silicon atoms, respectively; (**b**) system energy with the distance between TiO_2_ cluster and silicon surface.

In ultraviolet induced nanoparticle colloid jet machining, the interaction between the impacting nanoparticles and the workpiece surface are of important influence for the removal of workpiece materials. Utilizing the above calculation results, the interaction process between the TiO_2_ cluster and the hydroxyl silicon workpiece surface was calculated. Figure 7 shows the process of incident impacting TiO_2_ cluster approaching to silicon surface. According to the relationship between the system energy and the approaching distance shown in Figure 7b, when the TiO_2_ cluster is approaching the Si surface, a repulsive interaction exists permanently between the TiO_2_ cluster and the Si surface. The system energy significantly increases when the adsorption distance ranges from 2.0 Å to 1.3 Å. The main reason is that the two oxygen atoms of the OH groups adsorbed on TiO_2_ cluster and Si surface are infinitely close to each other, and the nuclear–nuclear repulsion between the nuclei of the two oxygen atoms makes the system energy significantly increased. Between the adsorption distance of 2.0 Å and 1.3 Å, the OH group on TiO_2_ cluster will impact with OH group on Si surface, and two atomic nuclei collide with each other. When the atomic orbits of two atoms overlapping, a covalent bond may be formed to reduce the energy of the system.

**Figure 8:**
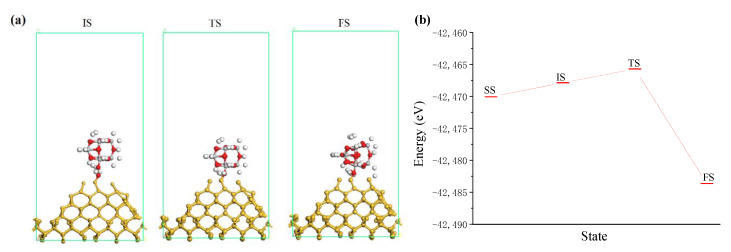
The interaction between incident TiO_2_ cluster and silicon surface. (**a**) Interaction states where white, red, grey and yellow balls are hydrogen, oxygen, titanium and silicon atoms, respectively; (**b**) system energy of interaction states.

Figure 8a shows the interaction between the incident TiO_2_ cluster and the silicon surface in the ultraviolet induced nanoparticle colloid jet machining, in which new chemical bonds (Ti-O-Si bonds) and products (a chemically adsorbed water molecule H_2_O) are generated. The supposed process can be depicted as follow:

A—OH + OH—B → A—O—B + H_2_O (Ch), (1)

where A represents TiO_2_ cluster and B represents Si atoms on the workpiece surface, H_2_O (CH) is a chemically adsorbed water molecule.

According to the results of Figure 8b, taking the energy of the separated state (SS) as the reference value, the adsorption energies of initial state (IS) and FS are −2.199 eV and 13.568 eV, respectively. The adsorption energy of the saddle point (TS) on the potential energy surface between the two states is −4.360 eV. From the IS state, the activation energy of the reaction is −2.161 eV, which is an endothermic reaction. In the process of ultraviolet induced nanoparticle colloid jet machining the kinetic energy of high speed incident nanoparticles can provide energy for the endothermic reaction to make the reaction proceed smoothly.

**Figure 9:**
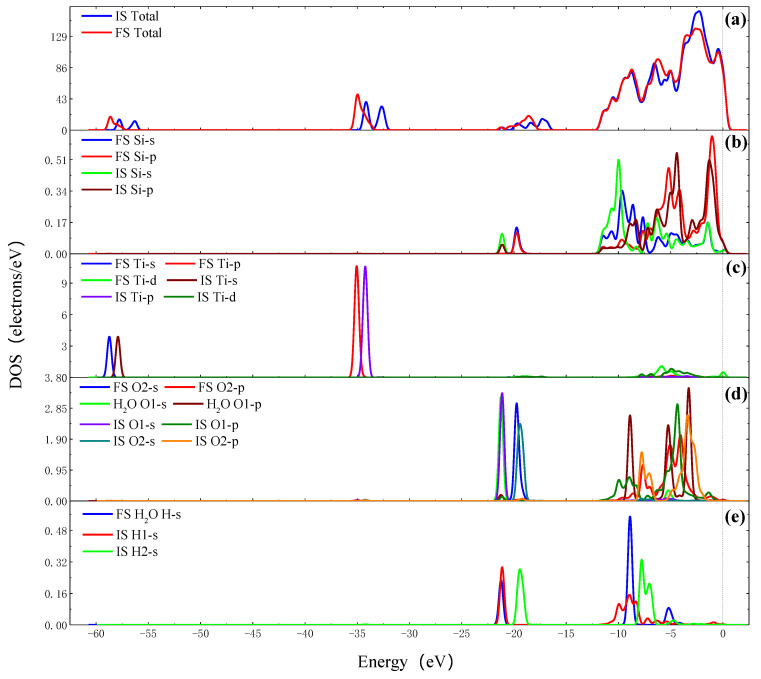
The DOS of TiO_2_ cluster adsorbing on the Si surface. (**a**) Total DOS of FS and IS; (**b**) PDOS of target Si atoms of FS and IS; (**c**) PDOS of target Ti atoms of FS and IS; (**d**) PDOS of O1 and O2 atoms of FS and H_2_O molecule and IS; (**e**) PDOS of H1 and H2 atoms of FS and H_2_O molecule and IS.

Figure 9 shows the density of states of TiO_2_ cluster adsorbing on the Si surface. The DOS of interaction between TiO_2_ cluster and the Si surface were analyzed. The results show that the Si 3s and 3p orbits of the target Si atom shift to the right and the electron strength increases. The Ti 4s, 3p and 3d orbits of the target Ti atom shift to the left. The O 2s and O 2p orbits of the O2 atom shift to the left, the electron strength of the O 2s increases and the O 2p decreases. The O 2p orbit of the O1 atom shifts to the left and the electron strength increases. The O 2s and O 2p orbits of the O2 atom hybridize with the Si 3s, Si 3p, Ti 3p and Ti 4s orbits and form Ti-O-Si chemical bonds. The O 2s and O 2p orbits of the O1 atom hybridize with the H 1s orbits of H1 and the H2 atoms and form a H_2_O molecule. In the above process, the Si 2p, Ti 4s, Ti 3p and O 2p (O2 atom) orbits gain electrons, the Si 3s and O 2p (O1 atom) orbits lose electrons.

### 3.3. Separation of Titanium Dioxide Cluster from the Surface of Monocrystalline Silicon

**Figure 10:**
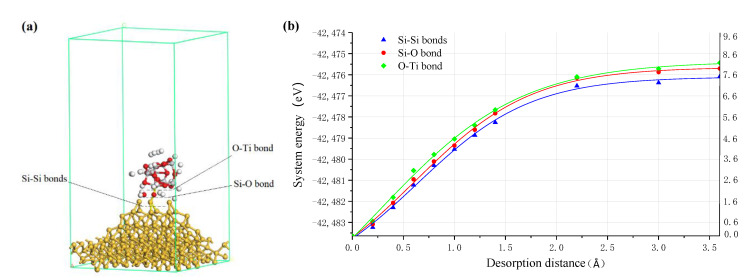
(**a**) Three kinds of bonds between TiO_2_ cluster and the Si surface where white, red, grey and yellow balls are hydrogen, oxygen, titanium and silicon atoms, respectively; (**b**) energy curve of TiO_2_ cluster separating from Si surface.

In ultraviolet induced nanoparticle colloid jet machining, the shear viscosity of the high-speed flowing colloid results in the nanoparticles adsorbed on the workpiece surface being separated from the surface. There are three kinds of chemical bonds between TiO_2_ cluster and the Si surface, two Si-Si bonds, one Si-O bond and one O-Ti bond, as is shown in Figure 10a. Among them, when the Si-O bond and the O-Ti bond are broken, the TiO_2_ cluster will be separated from the Si surface without any material removal from the Si surface. Only when the two Si-Si bonds are broken will the target Si atom on the silicon workpiece surface be removed.

Figure 10b shows the relationship between the system energy and the bond length of the above three kinds of chemical bonds when the chemically adsorbed TiO_2_ cluster is separated from the Si surface. According to the results, when the separating distance is about 1.4 Å, the energy curves of the system become smooth. It can be considered that at this point, each target bond begins to be broken and the TiO_2_ cluster is separated from the Si surface. The calculation results showed that the order of energy needed to break the above three bonds is that: *E*_Ti-O bond_ > *E*_O-Si bond_ > *E*_2 Si-Si bonds_. In the process of the TiO_2_ cluster being separated from the silicon surface, the two Si-Si back bonds will be broken preferentially in the separation process. When the two Si-Si back bonds are broken, the target Si atom of the silicon surface is removed together with the TiO_2_ cluster, thus realizing the atomic level removal of the workpiece material.

**Figure 11:**
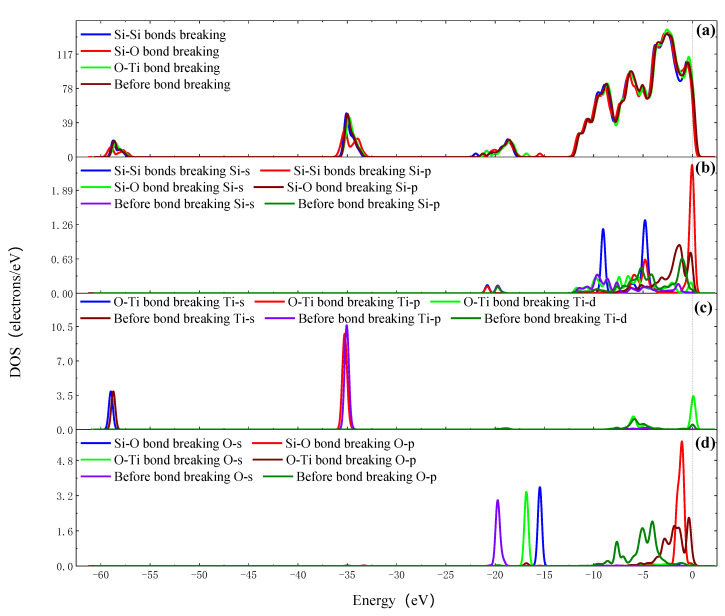
The DOS of TiO_2_ cluster separating from the Si surface. (**a**) Total DOS; (**b**) PDOS of adsorption site Si atoms; (**c**) PDOS of adsorption site Ti atoms; (**d**) PDOS of adsorption site O atoms.

Figure 11 shows the density of states of TiO_2_ cluster separating from the Si surface. Compared with the total DOS curve before bond breaking, because of the increase in the free bonds on the Si surface after the bond breaking of two Si-Si bonds—one Si-O bond and one O-Ti bond—there are more peaks in the total DOS curves when the TiO_2_ cluster is separating from Si surface with a distance of 1.4 Å. The Si 3s and 3p orbits of adsorption site Si atoms shift to the left, accompanying the electron strength increase after the two Si-Si bonds breaking and the electrons transferred from the Si 3p orbit to the Si 3s orbit. After the Si-O bond breaking, the Si 3s and 3p orbits shift to the right as well as the electron strength decrease, and the O 2s and 2p orbits shift to the right as well as the electron strength increase. The Si 3s, 3p and O 2s orbits gain electrons from the O 2p orbit in the process of the Si-O bond breaking. After the O-Ti bond breaking, the Ti 4s, 3p and 3d orbits shift to the left with the electron strength of Ti 3p decrease, and the O 2s and 2p orbits shift to the right as well as the electron strength increase. The O 2s and O 2p orbits gain electrons from the Si 3s, 3p orbits, and the Ti 4s, 3p and 3d gain electrons from the TiO_2_ cluster.

The above calculation model and results reflect the main bonding effects of the interface reactions between TiO_2_ nanoparticles and the silicon workpiece to remove material at the atomic level in ultraviolet induced nanoparticle colloid jet machining.

## 4. Results and Discussion

The instantaneous adsorption process of nanoparticles on the workpiece surface had been observed in previous study [24]. In order to verify the above calculation results experimentally, the experiment of ultraviolet induced nanoparticle colloid jet machining was carried out using the self-developed equipment to detect the adsorption of TiO_2_ nanoparticles on the silicon surface.

After 3 min of fixed-point injection of an ultraviolet induced nanoparticle colloid jet, the silicon workpiece surface was removed from the polishing table, soaked in deionized water and dried naturally at room temperature. Then, the morphology, microanalysis and chemical composition of the silicon workpiece surface were characterized by means of Scanning electron microscope (SEM) and X-ray photoelectron spectroscopy (XPS).

**Figure 12:**
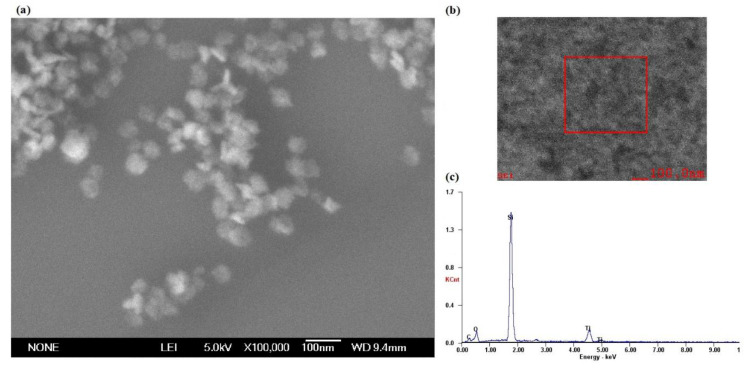
The morphology and microanalysis of TiO_2_ nanoparticles adsorbing on Si surface. (**a**) The morphology; (**b**) the microanalysis areas and (**c**) the microanalysis results.

Figure 12a shows the SEM morphology of the silicon surface on which the adsorbed TiO_2_ nanoparticles have been observed. The SEM results indicate that there are reunion adsorption phenomena of the TiO_2_ nanoparticles after the fixed-point injection of ultraviolet coupled nanoparticle colloid jet, which leads to the increases in particle size of the TiO_2_ nanoparticles adsorbing on the silicon surface. The reason for this phenomenon is that the polishing colloid used in the experiment has been reused many times to carry out relevant polishing experiments. Figure 12b,c shows the areas and results of scanning electron microscope energy spectrum (SEM-EDS) microanalysis of the silicon surface, respectively. The results indicate that the chemical composition of the silicon workpiece surface after fixed-point injection of ultraviolet coupled nanoparticle colloid jet is mainly composed of Si elements, as well as a small amount of Ti, O and C elements.

**Figure 13:**
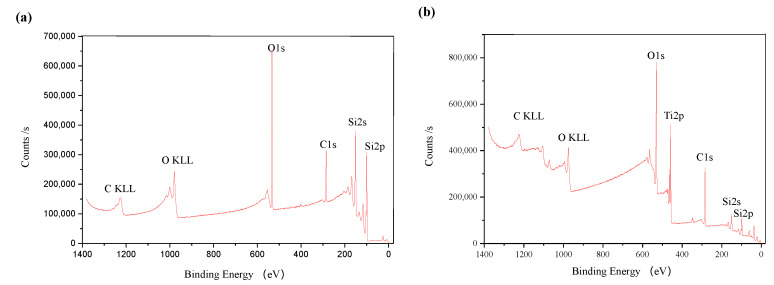
Full spectrum of (**a**) XPS of the original Si surface; (**b**) XPS of Si surface after TiO_2_ nanoparticle colloid jet impacting under ultraviolet irradiation.

X-ray photoelectron spectroscopy (XPS) was also used to analyze the silicon surface before and after TiO_2_ nanoparticle colloid jet impacting. Figure 13a,b shows the full spectrum of XPS of the original silicon surface and the silicon surface after TiO_2_ nanoparticle colloid jet impacting under ultraviolet irradiation, respectively.

Compared with the standard XPS spectrum, the elements on the original silicon surface are Si, C and O. Strong Si peak appears on the silicon surface, which is the main characteristic peak of the silicon surface. The existence of the O element is mainly due to the oxidation of silicon in contact with air. Since the detection depth of XPS is about 10 nm, and the surface layer is oxidized, the peak of O element in the full spectrum is obvious. There may be two reasons for the existence of C, one is the residual in the pre-process, and the other is that the sample surface is polluted by the environment due to too long a storage time. Si, C, O and Ti are the main elements of the silicon surface after TiO_2_ nanoparticle colloid jet impacting under ultraviolet irradiation. The weak Si peak and strong Ti peak appear in the spectrum, which indicates that the chemical adsorption of incident TiO_2_ nanoparticles on the silicon surface occur in the fixed-point injection of the ultraviolet induced nanoparticle colloid jet.

**Figure 14:**
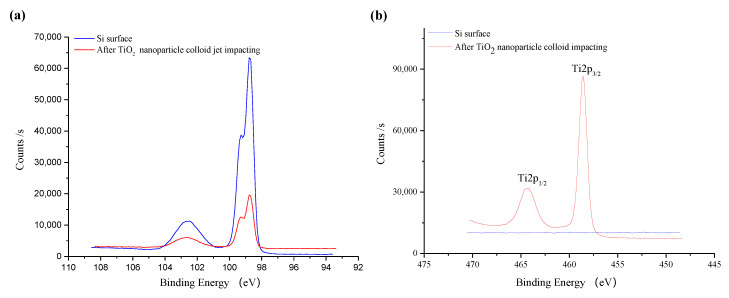

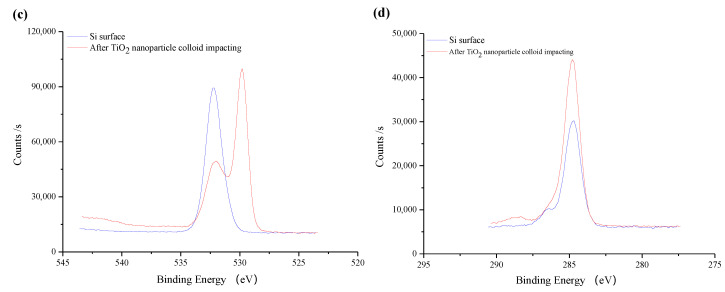
The fine spectrum of XPS on the silicon surface before and after TiO_2_ nanoparticle colloid jet impacting. (**a**) Si 2p scans; (**b**) Ti 2p scans; (**c**) O 1s scans; (**d**) C 1s scans.

Figure 14 shows the fine XPS spectrum of the original silicon surface before and after TiO_2_ nanoparticle colloid jet impacting. As is shown in Figure 14a, the fine spectrum of Si 2p is composed of two peaks, which are located near 99 eV and 103 eV [25]. The peak near 99 eV is significantly higher than the peak near 103 eV. Compared with the standard XPS spectrum, the binding energy of 99 eV is Si and 103 eV is SiO_2_. Therefore, the Si elements on both of the surfaces mainly existed in the state of Si and SiO_2_, and those in the form of the Si element are obviously more abundant than those in the form of SiO_2_. Due to the adsorption of TiO_2_, the content of Si detected on the surface after nanoparticle colloid jet impacting is relatively lower. Among them, the peaks of Si 2p on the original silicon surface are at 98.78 eV and 102.48 eV, and the peaks of silicon surface after nanoparticle colloid jet impacting are at 98.78 eV and 102.68 eV, the shift of 0.2eV to a high energy indicates that there may be chemical changes in the presence of electron transfer. According to the Figure 14b, there are no TiO_2_ nanoparticles on the original silicon surface. The fine spectrum of Ti 2p on the Si workpiece surface after nanoparticle colloid jet impacting is split into two levels due to the spin orbit coupling of electrons. The peak near 464 eV corresponds to Ti 2p 1/2, and the peak near 459 eV corresponds to Ti 2p 3/2 [26,27]. The peak position of Ti 2p 1/2 and Ti 2p 3/2 is 464.18 eV and 458.58 eV, respectively. The splitting value of standard TiO_2_ is 5.7 eV, while that of the Si workpiece surface after nanoparticle colloid jet impacting is 5.6 eV, which is 0.1 eV less than that of the standard TiO_2_. In Figure 14c, the O 1s peak on the original Si surface is 532.18 eV, which indicates that the presence of O element is mainly SiO_2_. The O 1s peak of 529.78 eV on the Si workpiece surface after nanoparticle colloid jet impacting belongs to TiO_2_, while the O 1s peak at 531.98 eV belongs to SiO_2_. However, the binding energy of O 1s on the Si workpiece surface shifts 0.2 eV to a low energy, relative to the original Si surface. Combining these results with the results of the Si 2p shift of 0.2 eV to a high energy, the O 1s shift of 0.2 eV to a low energy and the splitting value of TiO_2_ is 0.1 eV lower than the standard value. These results together indicate that new chemical bonds of Ti-O-Si are formed between TiO_2_ nanoparticles and the Si surface in the process of nanoparticle colloid jet impacting [28,29]. According to the results presented in Figure 14d, the C element exists on the surface of Si workpiece before polishing, which may be the residual in the pre-process. In the process of ultraviolet induced nanoparticle colloid jet impacting, new C elements are introduced to the Si workpiece surface by dispersant and surfactant in the colloid polishing solution.

The ultra smooth surface polishing experiment of the silicon workpiece was carried out on the ultraviolet induced nanoparticle colloid jet machining system with a polishing time of 120 min. The silicon workpiece surface was cleaned with deionized water under a pressure of 1 MPa to completely remove the remaining nanoparticles on the surface. Atomic force microscope (AFM, Brook, Dimension icon) was used to characterize the surface morphology and roughness of the workpiece before and after ultraviolet induced nanoparticle colloid jet machining with a measurement area of 10 μm × 10 μm.

**Figure 15:**
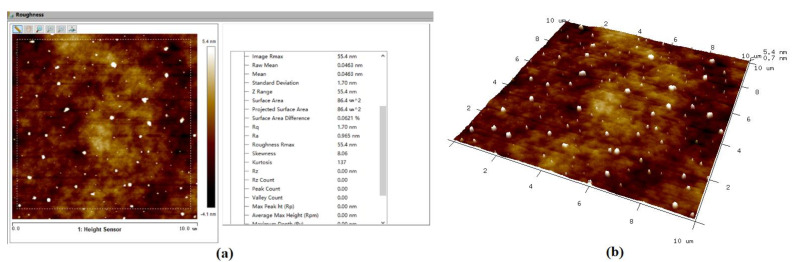
The original Si workpiece surface. (**a**) Surface roughness and (**b**) surface morphology.

**Figure 16:**
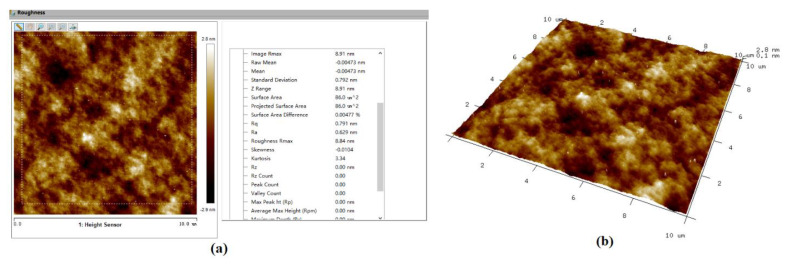
The Si workpiece surface after ultraviolet induced nanoparticle colloid jet machining. (**a**) Surface roughness and (**b**) surface morphology.

Figure 15 shows the AFM surface morphology and roughness results of Si surface before ultraviolet induced nanoparticle colloid jet machining. In the analysis area of about 86 μm^2^ the surface roughness is Rq 1.70 nm (Ra 0.965 nm). Combining with the AFM micrograph, a large number of surface protrusions, concave valleys and scratches left by the pre-process can be found on the original Si workpiece surface, and the maximum p-v value of surface protrusions and surface concave valleys is 55.4 nm. The AFM surface morphology and roughness results of the Si surface after ultraviolet induced nanoparticle colloid jet machining are shown in Figure 16. Within the same analysis area, the surface roughness is decreased to Rq 0.791 nm (Ra 0.629 nm). The surface protrusions, concave valleys and scratches are obviously removed and the maximum p-v value of the surface protrusions and surface concave valleys is reduced to 8.91 nm.

The above experimental results could support and verify the calculation results. Firstly, The SEM morphology and SEM-EDS results show that the adsorbed TiO_2_ nanoparticles had been observed on the Si workpiece surface after the fixed-point injection of an ultraviolet induced nanoparticle colloid jet, which was consistent with the calculated results of the TiO_2_ clusters adsorbing on the Si surface. Secondly, the results of the XPS analysis showed that new chemical bonds of Ti-O-Si were formed between the TiO_2_ nanoparticles and the Si surface in the process of nanoparticle colloid jet impacting, which further verified the possible reaction mechanism proposed in the calculation. Finally, in the ultra smooth surface polishing experiment the jet pressure was 1 MPa and the incident velocity of the nanoparticles was less than 30 m/s. At the impacting velocity, the incident energy of the 20 nm nanoparticle was too small to cause material deformation and material removal on the workpiece surface. Therefore, it could be considered that in ultraviolet induced nanoparticle colloid jet machining the surface material of the Si workpiece was removed by the chemical process shown in the previous calculation.

## 5. Materials and Methods

**Figure 17:**
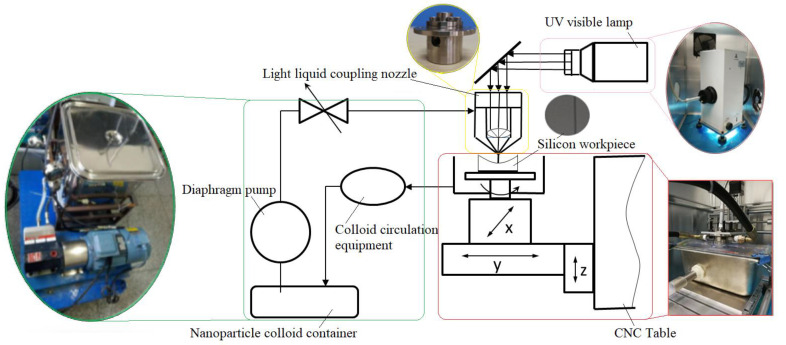
The system diagram of ultraviolet induced nanoparticle colloid jet machining.

The nozzle used in the experiments was a cosine type light liquid coupling nozzle. The injection pressure was 1 MPa, and the distance between nozzle and Si workpiece surface was 4 mm. Figure 17 shows the system diagram of ultraviolet induced nanoparticle colloid jet machining.

**Figure 18:**
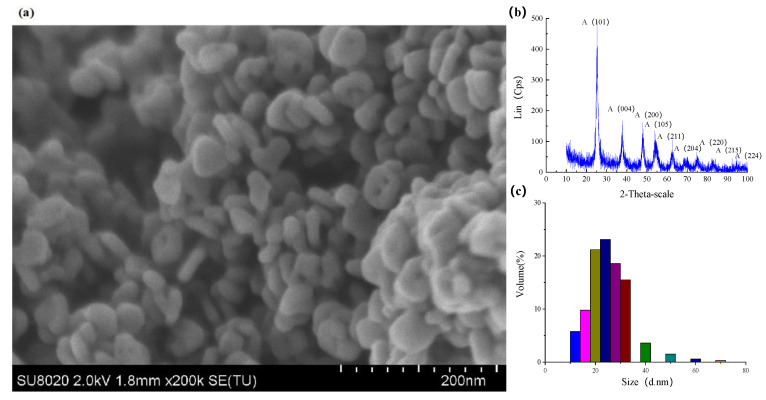
TiO_2_ nanoparticles used in the experiments. (**a**) SEM, (**b**) XRD pattern and (**c**) Diameter distribution.

In the experiment, the nanoparticles in the polishing colloid were anatase TiO_2_ with the volume concentration of 10% and pH value of 7. Figure 18a shows the SEM micrograph of TiO_2_ nanoparticles which was carried out on SU8020 high resolution field emission scanning electron microscope of HITACHI (Hitachi High-Technologies Corporation, Tokyo, Japan). Phase analysis of TiO_2_ nanoparticles was carried out on the D8 advance X-ray powder diffractometer (XRD, the X-ray source is Kα ray of Cu Target with wavelength of 0.15405 nm, the scanning speed is 12°/min, the scanning range is 20°~80° and the sampling interval is 0.02°, Bruker, Karlsruhe, Germany). Figure 18b shows the XRD pattern of TiO_2_ nanoparticles. Figure 18c shows the particle size distribution of the TiO_2_ nanoparticles measured by nanoparticle size analyzer (The Zetasizer Nano ZS particle size potentiometer of Malvern company, Malvern, UK, with a scanning speed of 1000/s). The diameter of the TiO_2_ nanoparticles were mainly distributed in the range of 20~30nm. Ultraviolet–visible light was provided by a 500 W high pressure mercury lamp with the intensity of 145 mW/cm^2^ measured by an optical power meter.

## 6. Conclusions

In this work, the atomic structure and electronic structure of the TiO_2_ cluster and the Si surface in the impacting process were studied based on the first principle calculations, to investigate the material removal mechanism of ultraviolet induced nanoparticle colloid jet machining. In the colloid, the OH groups easily adsorbed on the unpaired dangling bonds of the TiO_2_ cluster and the Si surface and formed stable surface adsorbed hydroxyl groups through exothermic processes. In the impacting process of the TiO_2_ cluster onto the Si surface, new chemical bonds of Ti-O-Si were generated through the chemical adsorption between the surface adsorbed hydroxyl groups of the TiO_2_ cluster and Si surface. At least 4.360 eV of energy was required to make the endothermic process proceed forward. Calculation results showed that the two Si-Si back bonds were the weakest bond among the three chemical bonds between the TiO_2_ cluster and the Si surface and were broken preferentially in the separation process of the TiO_2_ cluster from the silicon surface. The target Si atom was removed together with the TiO_2_ cluster and the atomic material removal of the workpiece surface was realized in ultraviolet induced nanoparticle colloid jet machining. A layer of adsorbed TiO_2_ nanoparticles was detected on the Si surface after 3 min of fixed-point injection of an ultraviolet coupled nanoparticle colloid jet by the scanning electron microscope. According to the X-ray photoelectron spectroscopy analysis results, the existing form of Si, O and Ti elements changed in chemical form after nanoparticle colloid jet impacting, which indicated that new chemical bonds of Ti-O-Si were formed. An ultra-smooth Si workpiece with surface roughness of Rq 0.791 nm (Ra 0.629 nm) was obtained and a large number of surface bulges and scratches were removed from the Si workpiece surface after 120 min of ultraviolet induced nanoparticle colloid jet machining.

## Data Availability

The data presented in the article are available from the corresponding author.

## Sample Availability

Samples of the colloid and workpieces are available from the corresponding author.
